# Ultrasound-Assisted Extraction of Protein from Pumpkin Seed Press Cake: Impact on Protein Yield and Techno-Functionality

**DOI:** 10.3390/foods11244029

**Published:** 2022-12-13

**Authors:** Deniz Sert, Harald Rohm, Susanne Struck

**Affiliations:** Chair of Food Engineering, Institute of Natural Materials Technology, Technische Universität Dresden, 01062 Dresden, Germany

**Keywords:** ultrasonic treatment, alkaline extraction, solubility, foaming stability, foaming capacity

## Abstract

Conventional solvent-based methods widely used for isolating plant proteins may deliver an unsatisfactory protein yield and/or result in protein degradation. The present study started with the optimization of pumpkin seed protein from press cake by alkaline extraction and subsequent isoelectric precipitation. Subsequently, extraction was supported by ultrasound under three conditions: ultrasonic treatment followed by alkaline extraction (US+AE), concomitant ultrasonic treatment and alkaline extraction (UAE), and alkaline extraction followed by ultrasonic treatment (AE+US). Compared to the control group, an increase in protein yield was achieved after ultrasonic treatment, while the highest protein yield was obtained with AE+US (57.8 ± 2.0%). Isolates with a protein content of 94.04 ± 0.77 g/100 g and a yield of 43.6 ± 0.97% were obtained under optimized conditions. Following ultrasonic treatment applied during extraction, solubility, foaming capacity, foam stability, and denaturation enthalpy of the isolated protein increased, and water binding capacity decreased as compared to non-sonicated samples. The d_90_ particle size percentile of the extracted suspensions was 376.68 ± 38.32 µm for the control experiments, and particle size was significantly reduced in ultrasound-assisted treatments down to d_90_ = 179.93 ± 13.24 µm for the AE+US treatment). Generally, ultrasonication resulted in a significant increase in protein yield and improved techno-functional properties of the isolates.

## 1. Introduction

The demand for food protein has increased in recent years as consumers become more aware of the need for a sufficient intake. As a consequence, the search for plant-based protein has become more intense [1], also because of critical aspects concerning animal proteins, such as animal welfare and high greenhouse gas emissions in industrial meat production [2,3]. Although residues of plant food production occur irregularly in the annual production cycle and require further processing, they can be considered prospective sources for protein extraction [4]. Using agro-industrial production losses for protein recovery might be a long-term solution that improves the economic potential of these by-products [5].

The oilseed press cake is a by-product of vegetable oil processing and contains a considerable amount of protein. Canola and sunflower seeds are the most popular and best-studied oilseeds [6], but pumpkin seeds can also be considered a valuable source of protein [7]. Pumpkin is a plant within the *Cucurbitaceae* family, mainly cultivated in Asia and several European regions. According to the Food and Agriculture Organization of the United Nations, worldwide pumpkin production reached over 27 million tons in 2020—63% from Asia, with China (approx. 7.5 megatons) being the largest producer [8]. Oil pumpkin is a special mutant variety with a tasteless and almost unpalatable flesh, exclusively cultivated for gaining the seeds, which are only covered by a thin membrane. A total annual seed harvest of approx. 75,000 tons obtained from ~100,000 hectares in Middle and Eastern Europe is used for the production of cold-pressed pumpkin oil with a yield of ~0.4 L/kg. The oil is sold at a price from 20 €/L upwards and, for a region in the southeast of Austria, registered as a “Protected Geographical Indication” (PGI) product (“Steirisches Kürbiskernöl”) by the European Union [9]. The popularity of pumpkin seeds for direct consumption is also progressively growing [10] since they are an excellent source of protein (content: 24.5–36 g/100 g), unsaturated fatty acids and secondary plant metabolites [11]. It is, for instance, recommended by the American Heart Association to consume approximately 30 g of pumpkin seeds per day due to its content of several nutrients showing positive effects on heart and bone health [12]. After removing residual oil from the pumpkin press cake, the protein content of the de-oiled fraction may increase to up to 65 g/100 g [13].

Extraction and precipitation techniques exhibit a significant impact on the structure and techno-functionality of the isolated protein and hence, on the applicability in processed foods. Water absorption capacity, amino acid composition, molecular shape and mass, net charge, size, solubility, isoelectric point, heat stability, hydrophobicity, and emulsification properties are just several of the important parameters that define the processing properties of plant proteins [3].

Ultrasound is a technology frequently used in protein extraction from plant-based sources [1,14,15]. Ultrasound-assisted extraction has several advantages, including improved yield, lower processing time, reduced solvent consumption, and minimal environmental influence [15,16,17]. Ultrasonic sound waves with a frequency of approx. 20 kHz usually result in severe cavitation effects. The energy released by the collapse of bubbles encourages the solvent to penetrate deeper into the suspended cell material, facilitating the mass transfer to and from the interface [18]. Studies using ultrasound support for protein extraction were, for instance, performed on barley [19], pea [20], peanut [1,21], canola [22], sunflower meal and seeds [16,23,24], rice bran [25,26], and walnut [27]. Several recent studies also showed that ultrasound application improves protein yield and functionality [19,21,28,29,30]. For instance, it was shown that ultrasound-assisted alkali extraction improves foaming and emulsifying capacity, solubility, and gel formation capacity [20,24].

Although there are many studies dealing with protein isolation from different plant sources, only little research is available on pumpkin seeds. The first aim of the current study was to improve the extraction of pumpkin seed protein from press cake by determining optimum parameters, with special emphasis on the protein content of the isolates and protein yield. Based on these preliminary findings, the effects of ultrasonic support during extraction on the physicochemical and techno-functional properties of the protein isolates were examined. Unlike previous research, the intention of our study was to assess the impact of ultrasound when introduced at different stages of the extraction procedure.

## 2. Materials and Methods

### 2.1. Materials

Milled press cake from pumpkin (*Cucurbita pepo* L. subsp. pepo var. styriaca GREB.) seed oil production was kindly provided by Estyria Naturprodukte GmbH (St. Ruprecht an der Raab, Austria). Prior to protein isolation, the fraction with a particle size of 300–600 µm was suspended in hexane at a ratio of 1:3 (*w*/*v*) for 40 min at room temperature while stirring at 500 rpm (MS-MP8, Witeg Labortechnik GmbH, Germany) [31]. After filtering the slurry through a Whatman #1 filter paper and repeating the hexane extraction twice, the de-oiled material was dried for 16 h at room temperature and stored at 6 °C until use.

### 2.2. Gross Composition Analysis

Protein and fat content, dietary fiber, moisture, and ash content of untreated and de-oiled pumpkin seed press cake powder were determined in duplicate using standard analytical techniques [32]. Protein content was determined by the Kjeldahl method (nitrogen conversion factor: 6.25), and fat content was determined by Soxhlet extraction with petroleum ether. Based on AOAC 991.43, dietary fiber was analyzed using an enzyme kit (Megazyme Ltd., Bray, Ireland). Moisture content was assessed by drying at 105 °C, and ash content was determined gravimetrically after incineration at 550 °C.

### 2.3. Protein Isolation

#### 2.3.1. Control Procedure

To identify the most appropriate conditions for the extraction of protein from milled press cake, dispersions in deionized water were adjusted to pH 8.0, 9.5, or 11.0 by using 0.5 N NaOH at a solvent-to-solid ratio of 10:1, 20:1, or 30:1, and then stirred at room temperature for 1 h, 2 h, or 3 h at 500 rpm on a magnetic stirrer. After centrifugation in a Biofuge Stratos (Thermo Scientific, Dreieich, Germany) at 8,000 rpm for 20 min, the supernatant was precipitated at pH 4.5, using HCl, and kept at room temperature for 1 h. After a second centrifugation step, the pellet was dissolved in a small amount of deionized water, pH was set to 7.0, followed by freeze drying (Beta 1–8 LDplus, Martin Christ Gefriertrocknungsanlagen GmbH, Osterode, Germany). The protein content of the isolate was determined by the Kjeldahl method, and protein yield calculated by equation [1] served as the second target variable:

(1) $$\mathrm{Protein}\;\mathrm{yield}\;\left(\%\right)=\frac{m_{i}\times c_{\mathrm{pi}}}{m_{s}\times c_{\mathrm{ps}}\;}\;\times \;100\;\%$$

*m*_i_ and *m*_s_ refer to the mass of the isolate and the initial sample, respectively. The protein content of the isolate is given by *c*_pi_, while the protein content of the initial sample is indicated by *c*_ps_.

To minimize the potential of unexpected deviation, the experimental Box–Behnken design contained 17 runs with five repetitions at the center point, and the response data of protein yield and protein content were analyzed using a quadratic model.

#### 2.3.2. Protein Extraction with Ultrasonic Support

Ultrasonic support during protein extraction from press cake was realized by using three approaches:

- ultrasonic treatment followed by alkaline extraction (US+AE),
- concomitant ultrasonic treatment and alkaline extraction (UAE), and
- alkaline extraction followed by ultrasonic treatment (AE+US).

**Figure 1:**
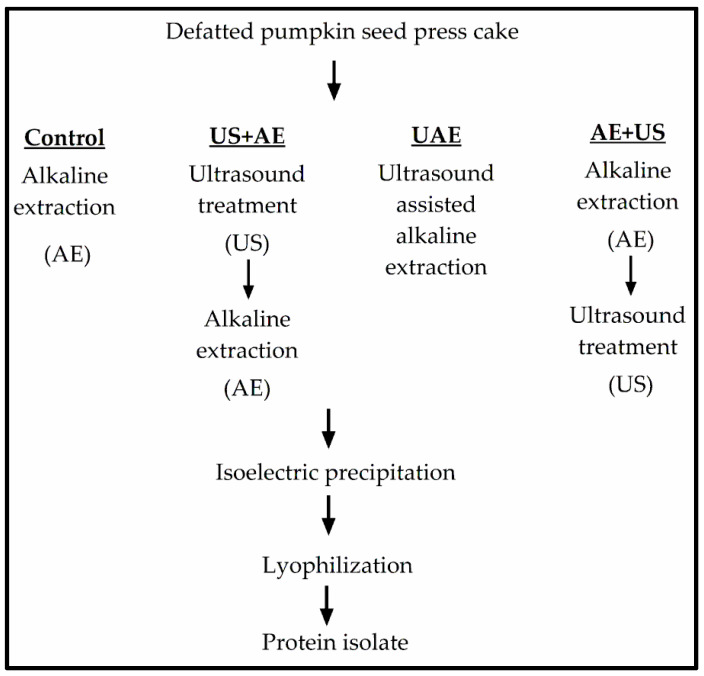
Outline of the protein isolation processes.

Ultrasonic treatments were carried out in four independent trials per condition, performed on two days (Figure 1), using a 25 kHz UDS 751/UP 200 S sonication unit with a 40 mm diameter sonotrode (Topas GmbH, Dresden, Germany) operated at a power density of 600 W/cm^2^ and 50% amplitude.

A solvent-to-solid ratio of 29, which was determined to be optimum in the preliminary control experiments, was applied in all treatments. Prior to ultrasonic support, dispersions were stirred on a magnetic stirrer at room temperature for 10 min at 500 rpm. In the US+AE treatment, pH was adjusted to the pre-determined optimum (pH 11) after 10 min of continuous ultrasonic application, and then the alkaline extraction was carried out for 60 min. In the UAE treatment, ultrasound was continuously applied for 10 min at optimum pH and solvent-to-solid ratio. In AE+US treatment, the alkaline extraction was carried out under the optimum conditions, followed by 10 min of continuous ultrasonic treatment. In every case, an ice bath was used to keep the temperature at 20 °C. All the treatments were followed by isoelectric precipitation and lyophilization of the isolate. The protein content of each isolate was determined in duplicate. The protein isolates obtained from the two runs performed on a single day were then pooled before further analysis.

### 2.4. Particle Size Distribution in Alkaline Extracts

Following one day of storage at 6 °C, the size distributions of the particles in the dispersions after alkaline extraction were measured by laser diffraction (Helos KR with Sucell, Sympatec GmbH, Clausthal–Zellerfeld, Germany) at pH 7.0. Measurements were carried out in the range of 10–15% optical density. After 2 min equilibration under stirring, size distributions were recorded, and the diameters at 10% (d_10_), 50% (d_50_), and 90% (d_90_) volume fractions were taken as particle size indicators. Analysis of each dispersion (four independent extractions per condition) was carried out in triplicate.

### 2.5. Determination of Techno-Functional Properties

#### 2.5.1. Protein Solubility

Protein solubility was determined using the method of Gonzalez–Perez [33] with modification. Each protein isolate (pooled samples) was dissolved in deionized water at a final concentration of 5 mg/mL, and solution pH was adjusted to 3, 5, 7, or 9 using 0.5 N NaOH or 0.5 N HCl. Prior to centrifugation at 6,000 rpm for 15 min, the respective solutions were agitated for 2 h at room temperature and 300 rpm. The Kjeldahl procedure was used to determine protein solubility, defined as the amount of protein in the supernatant (*p*_2_) related to the amount of protein in the initial sample (*p*_1_):

(2) $$\mathrm{Solubility}\;\left(\%\right)=\frac{p_{2}}{p_{1}}\times 100\%$$

#### 2.5.2. Water Binding Capacity

The method developed by Chen et al. [34] was used to determine the water binding capacity (WBC) of each pooled protein isolate in duplicate. Thirty mL of deionized water was added to 0.5 ± 0.01 g protein isolate and mixed for 30 s. After shaking in a horizontal position for 30 min at 20 °C, the mixture was centrifuged at 4000× *g* for 10 min. The dry matter of the supernatant was analyzed using an MA30 moisture analyzer (Sartorius AG, Göttingen, Germany) at 95 °C to determine the amount of soluble protein. WBC refers to the quantity of water bound per gram of dry protein that remains in the centrifuged sediment.

#### 2.5.3. Foaming Properties

To induce foam formation, deionized water was added to 0.5 ± 0.01 g of each protein isolate in duplicate, bringing the total volume to 50 mL (*V*_2_). The dispersion was homogenized for 2 min at 20,000 rpm using a T25 dispersion unit (IKA–Werke GmbH & CO. KG, Staufen, Germany). The samples were then immediately transferred into a measuring cylinder, where the total volume (*V*_1_) was measured. The foaming capacity was calculated according to Moure et al. [35]:

(3) $$\mathrm{Foaming}\;\mathrm{capacity}\;\left(\%\right)=\frac{V_{1}-V_{2}}{V_{2}}\times 100\%$$

The foam volume was determined at t = 0 min (*V*_0_) and after 10, 30, 60, 90, and 120 min (*V*_t_) to calculate foaming stability:

(4) $$\mathrm{Foaming}\;\mathrm{stability}\;\left(\%\right)=\frac{V_{t}}{V_{0}}\times 100\%$$

### 2.6. Different Scanning Calorimetry (DSC)

A Discovery DSC25 differential scanning calorimeter connected to an RCS90 cooling unit (TA Instruments, Eschborn, Germany) was used to record thermograms. Gallium and indium were used as standards for instrument calibration, and nitrogen at a flow rate of 50 mL/min served as purge gas. 

An empty standard aluminum pan served as control, and approx. 5 mg sample of each pooled protein isolate was weighed into the sample pan in duplicate. Following equilibration, the temperature was adjusted to 0 °C and then increased to 200 °C at a rate of 10 K/min. Measurements were made in duplicate. Peak denaturation temperature (*T*_den_) and denaturation enthalpy (∆*H*) were determined using the TRIOS 5.1.1 software.

### 2.7. Sodium Dodecyl Sulphate Polyacrylamide Gel Electrophoresis

The isolates were subjected to SDS-PAGE using the Laemmli method [36]. A 2 mg/mL protein solution was prepared with deionized water and mixed with 2x Laemmli buffer at a 1:1 (*v*/*v*) ratio. The mixtures were then heated to 95 °C for 5 min. Subsequently, 10 μL of the mixture was loaded into the electrophoresis equipment (C.B.S. Scientific Company Inc., Del Mar, CA, USA) and run for 1 h at 100 V. The running gel was then stained with Coomassie brilliant blue.

### 2.8. Color Profile

The color of each pooled protein isolate was measured in triplicate using a Luci 100 spectral colorimeter equipped with a D65 xenon lamp using the 10° observer (Hach Lange GmbH, Düsseldorf, Germany). Equation [5] was used to calculate color difference ∆*E* between the control and protein extracted using ultrasonic support:


(5) $$\Delta E=\sqrt{{\left(\Delta L^{*}\right)}^{2}+{\left(\Delta a^{*}\right)}^{2}+{\left(\Delta b^{*}\right)}^{2}}$$

Apart from lightness L*, chroma C* and the hue angle *h*_ab_ were calculated for interpretation [37].

### 2.9. Statistical Analysis

The statistical significance of the effects of the extraction procedures on the properties of the protein isolate was evaluated using analysis of variance (ANOVA) and subsequent Duncan multiple comparison tests (SPSS Inc., Chicago, IL, USA). To set up the preliminary optimization procedure, Design Expert version 7.0.0 (State-Ease, Inc., Minneapolis, MN, USA) was used.

## 3. Results and Discussion

### 3.1. Composition of Pumpkin Press Cake

Apart from 4.73 g/100 g moisture and approx. 13 g/100 g residual oil, the native pumpkin seed press cake contained 60.24 g/100 g protein and significant amounts of dietary fiber and salts (Table 1). De-oiling with hexane reduced residual oil content to below 1 g/100 g. The moisture content of the de-oiled sample was similar to that of the untreated powder, and the fraction of all other components increased accordingly. The protein content of the de-oiled press cake was 68.68 g/100 g.

As indicated by the data, pumpkin press cake is rich in protein and may serve as an excellent protein source when compared to, e.g., chia, sesame, rape, flax, sunflower, or hemp [38,39]. Bučko et al. [38] also de-oiled pumpkin press cake with hexane and found a residual protein content of 63.5 g/100 g. In their study on pumpkin protein isolation, Vinayashree and Vasu [7] specified the protein content of seed flour as 35.18 g/100 g, which increased to 51.85 g/100 g after de-oiling. The fat content of untreated and de-oiled press cake is similar to that reported in the literature [40,41] and appears typical for pumpkin. The same is true for the amount of salts and dietary fiber [42,43,44].

### 3.2. Optimization of the Protein Extraction Procedure 

During optimizing the control extraction procedure, it turned out that pH had the highest impact on protein yield, with increased yield obtained after extraction at higher pH. Higher alkalinity generally improves extraction efficiency as protein solubility and the negative charge of the side amino groups of basic amino acids is significantly affected [45]. It was also shown for sunflower protein that, in the range of 2–10, extractability increased with increasing pH [46]. ANOVA provided the conditions for maximizing protein yield and protein content of the isolate. These conditions were a pH of 11, a solvent-to-solid ratio of 29, and a soaking time of 60 min, which was not different from previous research on other substrates [47,48], and resulted in a protein yield of 43.6 ± 1.0% and a protein content of the isolate of 94.04 ± 0.77 g/100 g. All subsequent isolation procedures comprising ultrasonic support were carried out under these conditions. Details of the optimization routine can be accessed from Supplementary Materials.

### 3.3. Effect of Ultrasonic Treatment on Protein Yield and Content

**Figure 2:**
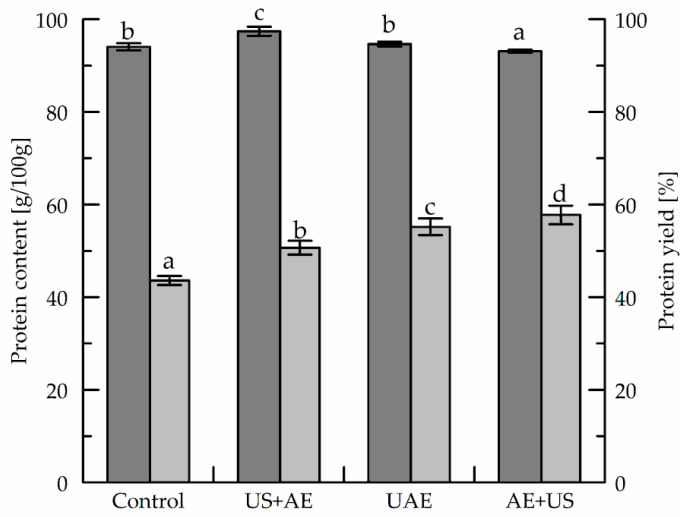
Protein content (dark bars) of dried pumpkin extract and protein yield obtained during extraction (light bars). Mean values ± standard deviations (*n* = 8) within the same bar type, labeled with different letters differ significantly (*p* < 0.05).

The effects of ultrasonic treatment on protein yield and protein content of the isolate are depicted in Figure 2. Compared to the protein yield of the control, the application of ultrasound under the given conditions (10 min, 50% amplitude) generally increased protein yield, which was 50.7 ± 1.5% with US+AE and 55.2 ± 1.8% with UAE. The highest protein yield was obtained with AE+US (57.8 ± 2.0%). However, enhanced acoustic cavitation might cause the denaturation of soluble proteins and hence reduce protein extraction efficiency [43]. In preliminary experiments with higher energy input (100% amplitude), we observed difficulties concerning temperature control but no significant increase in protein yield.

The highest protein recovery was achieved when the ultrasonic treatment followed alkaline extraction. Presumably, the underlying mechanical vibration intensifies the contact between the alkaline solution and the press cake powder, promotes cell destruction and opening of pores, and results in an improved mass transfer and, hence, protein release [16]. In a study on ultrasound-assisted protein extraction from cauliflower, protein content was 77.62 g/100 g, while extraction yield was 53.1% [49]. Another study used pulsed ultrasonic treatment at different power densities following alkaline extraction at pH 8.0 and room temperature for 1 h to extract protein from sunflower meal, and the protein yield was, depending on the respective conditions, 28.0–54.3% [16]. Tu et al. [43], who investigated ultrasonic extraction of albumin from de-oiled pumpkin seed using neutral extraction media, reported an extraction yield of 17.0%.

### 3.4. Particle Size Distribution of Alkaline Extracts

The extract produced using the control treatment showed the largest particles, and their size was reduced when ultrasound was applied (Table 2). This effect is caused by shear forces and cavitation; ultrasound affects electrostatic and hydrophobic interactions as well as hydrogen bonds and, as a result, decreases particle size [22,50]. In addition, the decrease in particle size was significantly (*p* < 0.05) more pronounced when alkaline extraction was followed by ultrasonic treatment (AE+US). Particle size reduction following ultrasonic treatment was also observed for canola protein isolate [22], pea protein isolate [20], sunflower protein isolate [16], and barley protein isolate [19]. As outlined in a study conducted by Malik et al. [24], the median particle size of sunflower protein isolates decreased from 114.6 µm to 94.3 µm after 10 min of ultrasonic treatment.

### 3.5. Protein Solubility

Solubility, counteracted by protein aggregation and denaturation, can be considered an indicator of protein functionality [14]. Table 3 shows that the solubility of the protein isolated from pumpkin press cake was largely affected by pH, showing a minimum (2.42 ± 0.06%) at pH 5. Generally, solubility is low near the isoelectric point but increases considerably at increased (pH ≤ 3.0) or reduced (pH ≥ 7.0) acidity because of the absence of electrostatic repulsive forces at the isoelectric point, resulting in protein molecules with neutral charge [51].

The results are consistent with the data of Vinayashree et al. [7], showing a pumpkin protein solubility of <10% at pH 4. Chavan et al. [52] observed that protein from beach pea had its lowest solubility at pH 4.5, and Dabbour et al. [16] reported sunflower protein solubility as low as 0.39% at pH 5, which is close to the isoelectric point of pH 4.5.

It was already shown that ultrasonic treatment improves protein solubility [14,27,53]. Compared to the control, the solubility of protein obtained by additionally applying ultrasound was significantly higher at each pH (see Table 3). This might be because proteins form aggregates in their native state, and cavitation is a physical factor that reduces hydrophobic interactions, necessary for the intermolecular association of protein molecules. It was also reported that ultrasound improves solubility by promoting the formation of monomers from insoluble or soluble protein aggregates, by affecting non-covalent interactions, and by releasing polar residues [23,53,54].

The AE+US treatment resulted in the highest protein solubility, presumably because of the impact of alkalinity on protein–water interactions prior to ultrasonic application as well as because of the changes in particle size. Ultrasonic treatment of soy protein isolates decreased particle size [14], and a protein solubility increase after ultrasonic treatment was also demonstrated for sunflower protein isolates [24].

### 3.6. Water Binding and Foaming Capacity

The effect of ultrasound on water-binding capacity is also shown in Table 3. When applying ultrasound during or after alkaline extraction, the WBC of the isolated protein was significantly lower. Considering conformational changes in protein molecules after an ultrasonic treatment and the subsequent formation of more soluble protein aggregates, it is likely that changes in the hydrophobic surface area are responsible for these differences. In a study on ultrasound-assisted sunflower protein isolation, an increase in protein solubility but a decrease in WBC was explained by the changes in surface hydrophobicity [24].

Foaming capacity (FC) refers to a protein’s ability to unfold quickly and to dissolve, forming a cohesive layer that surrounds gas bubbles, whereas foaming stability (FS) refers to the ability to generate stable foams by forming a continuous intermolecular polymer network that envelopes air cells [5]. Mechanisms such as rearrangements, penetration, and molecular movement at the interface significantly contribute to FC and FS.

**Figure 3:**
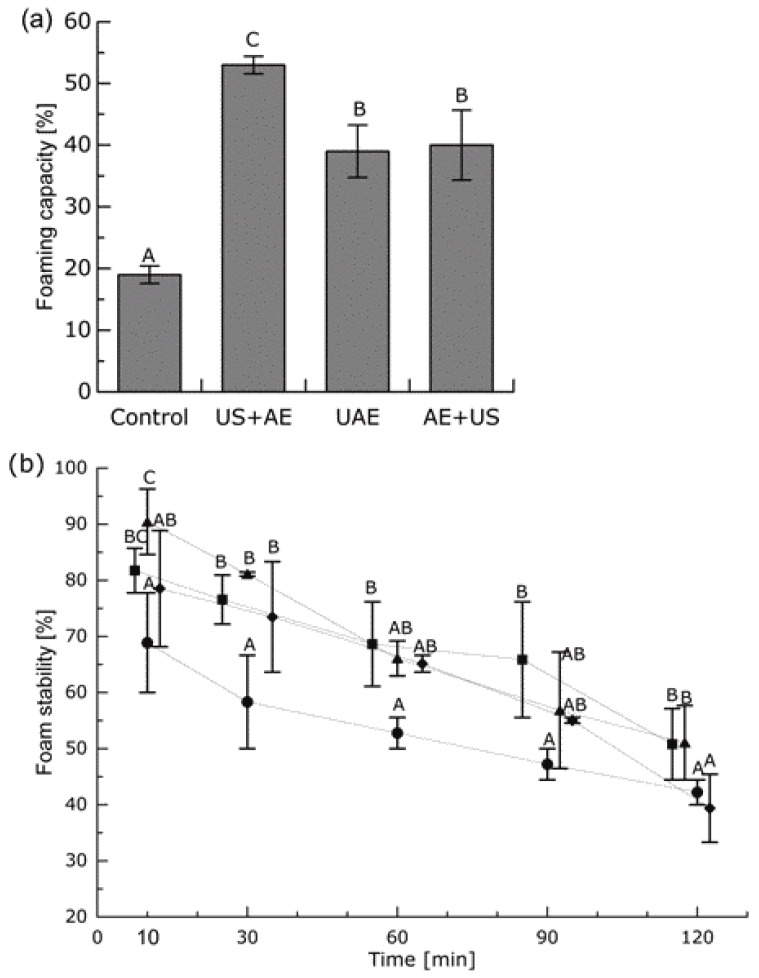
Foaming capacity (**a**) and foam stability (**b**) of protein isolates. Control (circles), alkaline extraction; US+AE (triangles), ultrasonic treatment followed by alkaline extraction; UAE (squares), ultrasonic treatment combined with alkaline extraction; AE+US (rhomboids), alkaline extraction followed by ultrasonic treatment. For better eye guidance, symbols are connected by dotted lines and are slightly shifted along the *x*-axis. Mean values ± standard deviations (*n* = 4) for the same period labeled with different letters differ significantly (*p* < 0.05).

Ultrasonic treatment caused a significant increase in FC (Figure 3). Due to structural unfolding, FC largely depends on protein diffusivity at the gas–liquid interface, and an increase may be related to the fact that ultrasound enhances dispersibility during foam generation by mechanically homogenizing the protein particles. Ultrasound also induces partial structural changes in proteins, leading to more rapid protein adsorption at the gas–liquid interface and, consequently, to an increased foaming capacity [23]. In studies with sunflower [24] and soy protein isolate [55], an FC increase was attributed to particle size reduction caused by ultrasound. This might also be the case for the pumpkin protein extracted in our study. According to Xiong et al. [29], pea protein interaction was improved by ultrasonic treatment, and FC increased from 58% to 73.3%. In a study conducted on protein isolation from evening primrose by-products, ultrasound-assisted alkaline extraction also increased the foaming capacity [56].

The foaming stability of pumpkin protein isolate also increased when ultrasound was applied (see Figure 3). Li et al. [57] reported that ultrasonic treatment enhanced the FS of brewer’s spent grain protein by 26.6% compared to the conventional extraction. It has been mentioned that ultrasound improves molecular flexibility, lowers surface tension, and facilitates the creation of strong elastic films around dispersed gas bubbles [58]. Ultrasonic support also increased the FS of sunflower protein isolates [24]. In the case of pea proteins, a smooth film at the air–water interface was responsible for the enhanced foam stability [29].

### 3.7. Stability of the Proteins

Table 4 summarizes the effects of the different extraction procedures on the thermal properties of the extracted protein. Protein denaturation temperature was the highest after control alkaline extraction, and significantly lower after the application of ultrasound, with the lowest *T*_den_ = 85.70 °C obtained in the US+AE treatment.

These findings demonstrate that ultrasonication caused structural changes of the proteins that resulted in a change in their thermal behavior. The decrease in *T*_den_ after ultrasound application confirmed the loss of protein–protein bonds. A reduction in denaturation temperature because of conformational changes was also determined in the studies where sunflower protein was extracted using ultrasound [24], and similarly during the isolation of soy protein [59].

The energy needed to denature or unfold protein structure is also related to the enthalpy required for this process. ∆*H* was 308. 6 J/g for the control sample and increased by approx. 20% when ultrasound was applied during protein extraction. The reason for this might be protein aggregation after prolonged sonication. Sonication of whey protein concentrates for up to 5 min resulted in a decline in enthalpy, implying the destruction of protein bonds. However, after more than 5 min sonication, the enthalpy increased, also indicating the possibility of re-aggregations [60].

The extracted pumpkin seed protein had a main molecular mass of approx. 35 kDa. Ultrasonic treatment did not cause significant differences in the electrophoresis profiles compared to the control treatment, demonstrating that the application of ultrasound does not modify the main structure of the protein. Similar results were observed in SDS-PAGE analyses of pumpkin seed protein isolate [30], peanut protein isolate [21], pea protein isolate [29], and soybean protein isolate [59,61].

### 3.8. Color Properties

Table 5 illustrates the color properties of the isolates, which were significantly different (*p* < 0.05). Ultrasound affects color as a result of cavitation. It could have a favorable or unfavorable impact on the color pigments in foods. While it may accelerate a structural release of the pigments, it can also induce light absorption by causing changes in the pigment-containing structures [28]. The color difference of the protein isolates was most significant after applying the AE+US treatment (2.72 ± 0.14).

There have been limited studies on changes in protein color caused by ultrasonication. A study conducted by Chittapalo and Noomhorm [62] on de-oiled rice bran demonstrated that sonicated rice bran protein concentrate was lighter. In case of protein extracted from album seed, ultrasonication also resulted in a higher lightness [28]. The hue angle expresses color quality, and 0° < *h*_ab_ < 90° indicates that protein isolate color is in the red–yellow quadrant of the L*a*b* color space. The increase in *h*_ab_ after ultrasound-assisted extraction indicates increased contributions of the yellow part of the spectrum. Chroma as a measure of color saturation was hardly affected by the impact of cavitation.

## 4. Conclusions

In this study, optimum alkaline extraction parameters (pH, solvent-to-solid ratio, and time) for protein extraction from pumpkin seed press cake were determined at pH 11, solvent-to-solid ratio of 29, and extraction time of 60 min. This research showed that the use of ultrasonication in combination with alkaline extraction enhances the protein yield. The protein recovery yield was 50.7 ± 1.5% with the US+AE and 55.2 ± 1.8% with the UAE, while AE+US provided the highest protein yield at 57.8 ± 2.0%.

The solubility of the protein obtained by applying ultrasound was considerably higher than the control and the highest when the AE+US treatment was applied (46.93 ± 0.61% at pH 9). Foaming capacity and stability significantly increased after ultrasonic treatment, and the highest foaming capacity was observed after using the US+AE treatment (53.0 ± 1.41%). Protein denaturation temperature decreased after ultrasonic treatment, as did the water binding capacity. In addition, ultrasound showed a significant impact on the size of the particles in the suspension.

Ultrasound is a promising and excellent alternative for assisting alkaline extraction of pumpkin press cake, and the protein obtained had appropriate techno-functional properties. When compared to the control, ultrasonic treatments increased solubility, foaming capacity, and stability while decreasing water binding capacity and particle size. The AE+US treatment was considered to be the most effective treatment among the three different ultrasonic treatments because it provided the highest increase in protein yield as well as high impacts on techno-functional properties. It can be assumed that protein yield and functionality can further be improved when ultrasound conditions are varied with respect to power input and treatment time.

## Figures and Tables

**Table 1:** Composition of untreated and de-oiled pumpkin seed press cake.

Component (g/100 g)	Untreated Press Cake	De-Oiled Press Cake
Moisture	4.73 ± 0.32 ^a^	4.84 ± 0.19 ^a^
Fat	13.38 ± 0.12 ^a^	0.77 ± 0.14 ^b^
Protein	60.24 ± 0.05 ^a^	68.68 ± 0.13 ^b^
Total dietary fibre	13.13 ± 0.71 ^a^	17.39 ± 0.54 ^b^
Ash	8.02 ± 0.06 ^a^	9.11 ± 0.07 ^b^

Mean values ± half deviation range (*n* = 2) in a row labeled with different letters differ significantly (*p* < 0.05).

**Table 2:** The particle size of dispersions after alkaline extraction.

Treatment	d_10_ [µm]	d_50_ [µm]	d_90_ [µm]
Control	4.82 ± 0.44 ^b^	72.12 ± 14.58 ^b^	376.68 ± 38.32 ^c^
US+AE	3.49 ± 0.20 ^a^	31.50 ± 2.51 ^a^	252.96 ± 24.85 ^b^
UAE	3.38 ± 0.25 ^a^	27.59 ± 4.02 ^a^	245.83 ± 34.60 ^b^
AE+US	8.05 ± 0.72 ^c^	36.25 ± 5.71 ^a^	179.93 ± 13.24 ^a^

US+AE, ultrasonic treatment followed by alkaline extraction; UAE, ultrasonic treatment combined with alkaline extraction; AE+US, alkaline extraction followed by ultrasonic treatment. d_10_, d_50_ and d_90_ refer to particle diameters at 10%, 50% and 90% volume fraction. Mean values ± standard deviations (*n* = 12) in a column labeled with different letters differ significantly (*p* < 0.05).

**Table 3:** Solubility and water binding capacity (WBC in g per g dry matter) of extracted pumpkin seed protein.

Treatment	Solubility [%]				WBC [g/g dm]
	pH 3	pH 5	pH 7	pH 9	
Control	35.73 ± 1.42 ^a^	2.42 ± 0.06 ^a^	15.17 ± 0.20 ^a^	37.82 ± 1.07 ^a^	3.95 ± 0.04 ^b^
US+AE	37.33 ± 0.41 ^b^	3.83 ± 0.42 ^b^	18.72 ± 0.13 ^b^	39.88 ± 0.30 ^b^	3.81 ± 0.08 ^b^
UAE	38.00 ± 0.89 ^bc^	6.59 ± 0.99 ^d^	18.80 ± 0.52 ^b^	46.87 ± 0.17 ^c^	3.61 ± 0.21 ^a^
AE+US	38.84 ± 0.21 ^c^	4.90 ± 0.05 ^c^	23.07 ± 0.06 ^c^	46.93 ±0.61 ^c^	3.66 ± 0.15 ^a^

US+AE, ultrasonic treatment followed by alkaline extraction; UAE, ultrasonic treatment combined with alkaline extraction; AE+US, alkaline extraction followed by ultrasonic treatment. Mean values ± standard deviations (*n* = 4) in a column labeled with different letters differ significantly (*p* < 0.05).

**Table 4:** Thermal properties of pumpkin seed protein isolates.

	Control	US+AE	UAE	AE+US
*T*_den_ (°C)	99.05 ± 4.15 ^c^	85.70 ± 2.29 ^a^	94.20 ± 2.84 ^b^	92.44 ± 3.58 ^b^
∆*H* (J/g)	308.6 ± 9.6 ^a^	371.0 ± 21.6 ^b^	362.2 ± 10.5 ^b^	360.8 ± 2.6 ^b^

*T*_den_, denaturation temperature; ∆*H*, enthalpy. Control, alkaline extraction; US+AE, ultrasonic treatment followed by alkaline extraction; UAE, ultrasonic treatment combined with alkaline extraction; AE+US, alkaline extraction followed by ultrasonic treatment. Mean values ± standard deviations (*n* = 4) in a row labeled with different letters differ significantly (*p* < 0.05).

**Table 5:** Color properties of pumpkin seed protein isolates.

	Control	US+AE	UAE	AE+US
∆E*		2.02 ± 1.26	1.84 ± 0.34	2.72 ±0.14
L*	71.20 ± 0.48 ^b^	72.26 ± 1.76 ^c^	69.64 ± 0.10 ^a^	68.78 ± 0.17 ^a^
*h*_ab_	78.70 ± 0.10 ^a^	80.33 ± 1.19 ^b^	81.05 ± 0.33 ^bc^	81.52 ± 0.08 ^c^
C*	21.66 ± 0.38 ^b^	20.57 ± 1.04 ^a^	22.08 ± 0.51 ^b^	21.29 ± 0.97 ^ab^

∆E*, color difference; L*, lightness; *h*_ab_, hue angle; C*, chroma. Control, alkaline extraction; US+AE, ultrasonic treatment followed by alkaline extraction; UAE, ultrasonic treatment combined with alkaline extraction; AE+US, alkaline extraction followed by ultrasonic treatment. Mean values ± standard deviations (*n* = 6) in a row labeled with different letters differ significantly (*p* < 0.05).

## Data Availability

The data that support the conclusions of this study are available upon reasonable request from the corresponding author.

## Supplementary Materials

Information concerning the optimization procedure for the extraction of pumpkin seed protein can be downloaded at: https://www.mdpi.com/article/10.3390/foods11244029/s1, Protein Extraction Optimization.
