# Supplementary material for: Ultrasound-Assisted Extraction of Protein from Pumpkin Seed Press Cake: Impact on Protein Yield and Techno-Functionality
Source: Foods. 2022 Dec 13;11(24):4029. doi: 10.3390/foods11244029 (PMC9777787; doi:10.3390/foods11244029)
Supplement: Supplementary file 1 [file foods-11-04029-s001.zip › foods-2080063-supplementary.pdf]

Information Concerning the Optimization of the Protein Extraction Procedure

The following table summarizes the levels of the independent variables X_1 (pH), X_2 (solvent-to-solid ratio) and X_3 (extraction time, all in coded and uncoded form) for optimizing pumpkin protein extraction from the press cake, and the resulting effects on protein yield (Y_1) and protein content of the isolate (Y_2). Protein yield, i.e., the fraction of protein transferred from press cake to isolate, and its protein content ranged from 0.5 to 39.6 % and from 93.19 to 99.66 g/100 g, respectively.

**Coded variables**			**Uncoded variables**			**Response**	
X_1	X_2	X_3	X_1: pH	X_2: Solvent-to-solid ratio (mL/g)	X_3: Time (h)	Y_1: Protein yield (%)	Y_2: Protein content (g/100 g)
-1	-1	0	8.0	10	2	0.5	94.47
-1	0	-1	8.0	20	1	0.9	97.22
-1	0	+1	8.0	20	3	3.3	93.19
-1	+1	0	8.0	30	2	3.3	96.92
0	-1	-1	9.5	10	1	3.1	98.10
0	-1	+1	9.5	10	3	6.8	98.42
0	0	0	9.5	20	2	16.8	98.58
0	0	0	9.5	20	2	19.0	99.66
0	0	0	9.5	20	2	21.0	98.30
0	0	0	9.5	20	2	17.9	98.36
0	0	0	9.5	20	2	17.0	98.65
0	+1	-1	9.5	30	1	20.1	98.79
0	+1	+1	9.5	30	3	23.7	99.64
+1	-1	0	11.0	10	2	21.7	98.25
+1	0	-1	11.0	20	1	34.7	99.45
+1	0	+1	11.0	20	3	33.9	99.21
+1	+1	0	11.0	30	2	39.6	99.26

By using the coded variables, the second order model for protein yield comes with a coefficient of determination of R^2 = 0.97, and pH (X_1), solvent-to-solid ratio (X_2), $X_1 X_2$, X_1^2 and X_2^2 were the model's significant variables (P < 0.05):

$$Y_2\ (\%) = 17.70 + 15.23 \cdot X_1 + 6.81 \cdot X_2 + 3.77 \cdot X_1 \cdot X_2 + 1.29 \cdot X_1^2 - 3.49 \cdot X_2^2$$

Factor X_1 (pH) had the highest impact on protein yield, indicating that protein yield increased significantly with increasing extraction pH. Protein content provides information on isolate purity. The respective model equation comes with one significant ($p < 0.05$) factor, namely pH in the linear and in the quadratic term (R^2 = 0.70):

$$Y_2\ (\mathrm{g\ 100\ g^{-1}}) = 98.72 + 1.80 \cdot X_1 - 1.48 \cdot X_1^2$$

In a final step, ANOVA provided the conditions to be used for maximising Y_1 and Y_2. The calculated values are pH 10.99 (changed to 11.0), a solvent-to-solid ratio of 28.8 (changed to 29), and a soaking time of 61 min (changed to 60 min), giving a protein yield of 43.6 ± 0.97% and a protein content of the isolate of 94.04 ± 0.77 g/100 g.
